# Street Ambulances and Accidents in the Metropolis

**Published:** 1907-02-16

**Authors:** 


### STREET AMBULANCES AND ACCIDENTS IN THE METROPOLIS.

### AMBULANCES AT PRESENT IN USE IN LONDON.

In view of the fact that a Departmental Committee appointed by the Home Secretary is now considering the existing provisions for dealing with street accidents in London, exceptional interest is attached to a conference of delegates from Metropolitan Boards of Guardians and other authorities held at Gray's Inn on Saturday.

#### The Ambulances Exhibited.

The delegates had an opportunity of inspecting thirty examples of ambulance cars sent by many of the London authorities and societies interested in ambulance work.

The Guardians of St. Olave's have adopted a simple and ingenious appliance for conveying patients down narrow staircases devised by Mr. D. Parkinson, master of Bermondsey Workhouse. It consists of a stout sheet of canvas with button-holed slits for the hands of the bearers, costing from 2s. to 4s. The idea is an excellent one and should recommend itself to all interested in the transport of the sick in large cities. A great number of Boards of Guardians still employ antiquated ambulance cars, that of St. George's being one of the worst. Fulham also has a one horse brougham pattern to do its Poor-law work. Hackney Union adheres to the same type. Lambeth has also an antiquated carriage. Wandsworth Guardians still employ a converted brougham for ambulance purposes. The more modern and up-to-date vehicles for the transport of the sick were represented by that of the Holborn Guardians, built on the most modern principles by the St. John's Ambulance Association. This forms a type of the most modern transport wagons, which many of the other Boards of Guardians have adopted as a model. The Metropolitan Asylums Board exhibited modern types of ambulance wagons in many forms, and the Royal Army Military Ambulance Corps (London Companies) showed a complete equipment of horse ambulance for campaigning purposes. There were two motor ambulances, one from the Metropolitan Asylums Board, and the other built for the Corporation of the City of London. In this ambulance the stretchers are of wood, free from projections and having the minimum of metal-work about them. This is owing to their having continually to be washed with disinfectants which are apt to corrode any metallic parts. An air-bed is placed on the stretcher to minimise vibration and to prevent jolting. It can be readily sponged over with disinfectants. For non-infectious work a mattress and pillow stuffed with the best horse-hair are in use. A specimen of these was shown in the Westminster Union's ambulance. A waterproof sheet is placed on the mattress, and a washable linen cover over all. Ambulances are either of the van or brougham shape. The latter has the disadvantage of being able to carry only a single patient. The van ambulance carries two patients, one above the other. In the latest pattern of the M.A.B. van ambulance the use of lowering gear is obviated by a bed being placed on either side at the seat level. This is more comfortable for the patients and better for the nurse.

The Bischoffsheim Association showed one of the street boxes containing an ambulance litter on wheels, and appliances for first aid. The Electromobile Company, 7 Curzon Street, Mayfair, W., showed a motor ambulance built for the Corporation of the City of London. Wilson and Stockhall, Bury, Lancashire, showed a patent accident ambulance, for one or two horses. This is capable of carrying two patients with an attendant, and has specially patented arrangements for raising and lowering stretchers. The same firm exhibited a patent brougham ambulance for one recumbent patient. The body of this vehicle is hung on patent rubber ball-bearing springs. Carters, New Cavendish Street, Portland Place, W., had two-wheeled and three-wheeled litters on exhibition. The Metropolitan police brought with them the wheeled litter now in general use.

#### The Discussion.

The Lord Mayor said it was impossible to exaggerate the importance, interest, and urgency of the question which had brought them together. Where time was not an element there was no difficulty in obtaining ambulance service, but in emergency cases it was necessary that an ambulance system should be efficient, uncomplicated, and everywhere readily available to the general public. He advised the public to take advantage of first-aid classes, and to avail themselves of the admirable public work instituted by Mr. Bischoffsheim. He did not think these installations were sufficiently advertised. Some notices ought to be fixed to the lamp-posts here and there in the metropolis showing where the nearest Bischoffsheim station could be found. He would like to see in the metropolis a complete modern system combining the maximum of completeness, universality, and efficiency with the minimum of friction, over-lapping, and expense to the ratepayers.

Mr. Thomas Ryan, honorary secretary to the Bischoffsheim ambulance service, gave an account of the Bischoffsheim installation from its establishment in 1889, and mentioned the number of times it had been called into requisition since its establishment. He denounced the four-wheeled cab as a conveyance utterly unsuitable for injured persons. London required first-aid for sufferers and a suitable means of transport. First-aid involved men and a system of training. Transport required the means of locomotion, and men to use it. Wherever in the metropolis an accident happened, especially in its busiest parts, the victims fell first of all into the hands of the police. The Commissioner's latest report showed the number of accidents in the streets in one year to have been 11,860, of which 7,625 were conveyed to hospitals by the police, the remaining 4,478 being dealt with by other persons. The training of the police was not quite satisfactory. The means of transport usually employed was four-wheeled cabs. Occasionally police ambulances were used, but were utterly unsuitable for cases of accident, having been originally designed mainly for the transport of drunkards and incapables. He urged that the hand ambulance of the Bischoffsheim system was an ample provision for street accidents, and could be maintained at an average cost per ambulance of £10 per annum, whereas each horse ambulance cost £200, a sum greatly in excess of any advantage likely to result from its use and that the ambulance services of London could be co-ordinated.

Mr. H. Thompson Lyon, chairman of the ambulance of the Metropolitan Asylums Board, who presided, said that during the next few weeks the Commissioner of Police would place sixty or seventy police litters at the disposal of the public.

#### Resolutions Adopted.

Several resolutions were moved by Mr. Tasker, who represented the Guardians of St. George's, Hanover Square. They expressed the opinion that an effective service of ambulance could be established and maintained without expense to the ratepayers by co-ordinating and developing already existing services, and by empowering the Poor-law authorities to place the ambulances they possess at the disposal of the public, making a charge in cases where it might be thought advisable. They were eventually declared carried.

